# Hepatocyte Nuclear Factor 4 Alpha Is a Key Factor Related to Depression and Physiological Homeostasis in the Mouse Brain

**DOI:** 10.1371/journal.pone.0119021

**Published:** 2015-03-16

**Authors:** Kyosuke Yamanishi, Nobutaka Doe, Miho Sumida, Yuko Watanabe, Momoko Yoshida, Hideyuki Yamamoto, Yunfeng Xu, Wen Li, Hiromichi Yamanishi, Haruki Okamura, Hisato Matsunaga

**Affiliations:** 1 Department of Neuropsychiatry, Hyogo College of Medicine, Nishinomiya, Hyogo, Japan; 2 Laboratory of Tumor Immunology and Cell Therapy, Hyogo College of Medicine, Nishinomiya, Hyogo, Japan; 3 Laboratory of Neurogenesis and CNS Repair, Hyogo College of Medicine, Nishinomiya, Hyogo, Japan; 4 Section of Behavioral Science, Kouiken Co., Ltd., Akashi, Hyogo, Japan; 5 Hirakata General Hospital for Developmental Disorders, Hirakata, Osaka, Japan; Chiba University Graduate School of Medicine, JAPAN

## Abstract

Major depressive disorder (MDD) is a common psychiatric disorder that involves marked disabilities in global functioning, anorexia, and severe medical comorbidities. MDD is associated with not only psychological and sociocultural problems, but also pervasive physical dysfunctions such as metabolic, neurobiological and immunological abnormalities. Nevertheless, the mechanisms underlying the interactions between these factors have yet to be determined in detail. The aim of the present study was to identify the molecular mechanisms responsible for the interactions between MDD and dysregulation of physiological homeostasis, including immunological function as well as lipid metabolism, coagulation, and hormonal activity in the brain. We generated depression-like behavior in mice using chronic mild stress (CMS) as a model of depression. We compared the gene expression profiles in the prefrontal cortex (PFC) of CMS and control mice using microarrays. We subsequently categorized genes using two web-based bioinformatics applications: Ingenuity Pathway Analysis and The Database for Annotation, Visualization, and Integrated Discovery. We then confirmed significant group-differences by analyzing mRNA and protein expression levels not only in the PFC, but also in the thalamus and hippocampus. These web tools revealed that hepatocyte nuclear factor 4 alpha (Hnf4a) may exert direct effects on various genes specifically associated with amine synthesis, such as genes involved in serotonin metabolism and related immunological functions. Moreover, these genes may influence lipid metabolism, coagulation, and hormonal activity. We also confirmed the significant effects of Hnf4a on both mRNA and protein expression levels in the brain. These results suggest that Hnf4a may have a critical influence on physiological homeostasis under depressive states, and may be associated with the mechanisms responsible for the interactions between MDD and the dysregulation of physiological homeostasis in humans.

## Introduction

Major depressive disorder (MDD) is recognized as one of the most common mental disorders in developed countries, including Japan [1–2]. However, the etiological and psychobiological mechanisms responsible for the development of MDD remain unclear, even though pharmacological treatments for MDD have been investigated extensively. These treatments have primarily targeted deficiencies in monoamine neurotransmitters or receptors, particularly serotonin and noradrenergic systems [3].

Evidence of significant relationships between MDD and dysfunction in the prefrontal cortex (PFC) and hippocampus is growing. For example, reduced expression of synapse-related genes and loss of synapses has been reported in the PFC of patients with MDD [4–5]. Moreover, studies of MDD patients have revealed that certain types of depressive symptoms may be associated with a decrease in the integrity of the ventromedial PFC and cingulate cortices [4–6]. Stressful conditions exert critical effects on the PFC and limbic or prelimbic regions, resulting in hyperactivity in the hypothalamic–pituitary–adrenal (HPA) axis and hippocampus [7–8]. The hippocampus is a key limbic area that is located at the “cross-roads” of circuitry. It regulates stress responses by providing inhibitory feedback to the HPA axis and also plays a role in mood modulation and memory function. Although these findings support a close association between the PFC, thalamus, and hippocampus in MDD, the molecular mechanisms responsible for physiological aspects of MDD remain unknown [9–11].

Recent meta-analyses have detected significantly higher concentrations of the preinflammatory cytokines, tumor necrosis factor (Tnf)-alpha and interleukin (IL)-6, in MDD patients compared with normal controls [12,13]. Even though the HPA axis reduces inflammatory activity via cortisol production, continuous inflammatory activity causes development of glucocorticoid resistance, which in turn leads to excessive inflammation, one of the risk factors of depression [14]. Inflammatory cytokines such as Tnf-alpha activated indoleamine 2,3-dioxygenase (IDO) may be involved in the underlying biological mechanisms [15]. IDO is a rate-limiting enzyme in the tryptophan to kynurenine pathway, an alternative to the tryptophan to serotonin pathway [15]. IDO activated by cytokines decreases serotonin in the brain owing to a shift in tryptophan metabolism from serotonin to kynurenine [15]. This appears to coincide with emergence of depressive states in patients treated with corticosteroids or interferon therapy [16–18]. Moreover, coagulation dysfunction may also exert a causal effect on the development of MDD, as MDD often develops after cardiac or cerebral infarction, and anticoagulant treatments are effective in such cases, even alleviating comorbid MDD [19–21]. In contrast, MDD may have a crucial impact on the short- and long-term outcomes of these infarction diseases, with significantly higher mortality in patients with MDD [19]. Biological mechanisms of coagulation dysfunction may be influenced by a number of findings, including platelet reactivity activation in the depressed state, serotonergic antidepressant effects on blood coagulation factor VIII, and decreased von Willebrand factor due to acute anxiety [22–23]. Thus, MDD appears to be closely associated with physiological dysfunctions in hormonal activity, coagulation, and inflammation [16–17, 19]. Some clinical studies, have consistently shown that MDD is often comorbid with metabolic syndrome components such as visceral obesity, dyslipidemia, insulin resistance, and hypertension [24–26]. The prevalence of hyperlipidemia in patients with MDD has been reported to be higher than that in the general population [27]. Additionally, the average incidence of hyperlipidemia or diabetes in patients with MDD was also higher than in healthy people [24, 27–28]. Taken together, MDD is a disorder associated not only with psychological, socio-cultural, and neurobiological factors, but also other pervasive physical dysfunctions. Nevertheless, the mechanisms and pathogenesis responsible for the interactions between these factors and MDD have not yet been clarified.

Therefore, to clarify these issues, we generated depression-like behavior in mice in the present study using a chronic mild stress (CMS) model. Indeed, the CMS model has been well validated as an animal model of MDD [29–30]. However, its biological validity has not been fully examined. In particular, the physiological aspects of the CMS model, such as lipid metabolism, hormonal function, or coagulation, have not yet been verified. These factors are closely associated with the general health of people, and significant dysfunction of these factors is expected to cause a variety of physical problems, such as dyslipidemia or insulin resistance. These problems can significantly increase the risk of serious diseases such as diabetes, myocardial infarction or stroke [31–33]. In this study, therefore, we sought to examine the molecular mechanism of MDD, focusing on lipid metabolism, hormonal activity and coagulation, using the CMS model. In addition, we elucidated the functions of amine synthesis, including serotonin metabolism, and the immune system to analyze the correlations among these factors.

## Materials and Methods

### Animals

We purchased experimentally naive male C57BL/6N mice from Japan SLC, Inc. for use in the present study. Mice were 9–10 weeks old and weighed 22.3 g on average at the start of the experiment. All mice were housed in groups of 3–5 in polycarbonate cages that were placed in a colony room maintained at a constant temperature (22 ± 1°C) and humidity (50–60%), under a 12-h light/dark cycle (lights on at 7:00 am) with free access to food and water.

Animals were randomly assigned to one of two groups; 25 control mice (C group) were given ordinary daily care, whereas 25 chronic mildly stressed mice (CMS group) were housed individually and exposed to CMS for 4 weeks according to previously published procedures [34]. Sixteen mice from each group were used in the molecular and tail suspension tests, whereas the rest were used for the open space swimming test.

Animal experiments were conducted according to the “Guide for Care and Use of Laboratory Animals’’ published by the National Institutes of Health (NIH) and approved by the “Ethical committee of Behavioral and Medical Science Research Consortium” (Hyogo, Japan). The approved IDs were 2012-B-09 and 2012-B-10. All efforts were made to minimize the suffering of animals including the number of mice used for all analyses.

### CMS procedure

The CMS procedure consisted of various unpredictable mild stressors including water deprivation (8 h), continuous overnight illumination (36 h), a wet cage (200 ml water in 100 g sawdust bedding; 4 h), 45° cage tilt (8 h), physical restraint with a suitably designed mouse holder (4 h), forced swimming in a plastic cylinder (diameter, 18 cm; height, 30 cm) containing water (24°C) at a depth of 15 cm (30 min), and exposure to 60 inescapable electric shocks (0.36 mA, 1 s) presented on a variable interval 60-s schedule in a chamber with a grid floor (10 × 10 × 10 cm). These stressors were randomly scheduled over a 1-week period and repeated 4 times.

### Behavioral tests

Standardized behavioral tests were performed 4 weeks after the study was initiated to quantitatively compare behavioral differences between groups.

### Open space swimming test (OST)

The open space swimming test was conducted using a circular pool (inside diameter: 95 cm, depth: 35 cm) enclosed by white featureless walls (width: 130 cm, height: 120 cm). The pool was filled with water to a depth of 20 cm and made opaque by the addition of titanium oxide. The temperature of the water was maintained at 22 ± 1°C. Each mouse was placed in the pool, with its head facing the outer edge of the pool, and allowed to swim (or not swim) freely for 10 min. All trials were recorded with a digital video camera placed above the maze. The swim path distance was calculated by a computerized video-based tracking system (Be-Chase ver.3.0, Kouiken Co. Ltd., Akashi, Japan).

### Tail suspension test (TST)

The tail suspension test was performed using the Be-Sensor system (Taiyo Electric Co. Ltd., Osaka, Japan). An isolation chamber (45 × 45 × 45 cm) equipped with an infrared ray sensor on the ceiling was used. This sensor consisted of paired infrared pyroelectric detectors that measured heat energy radiating from the mouse. Each mouse was suspended by the tail using adhesive tape from a horizontal bar attached 30 cm above the chamber floor for 6 min. Struggling activity during tail suspension was quantitated by analyzing changes in heat energy.

### Sample collection

We euthanized the mice by decapitation using a guillotine, and collected brain samples for further analyses according to previously described methods [35]. Ten mice per group were used for microarrays and quantitative real-time polymerase chain reaction (qRT-PCR) and 6 were used for western blotting analysis (WB). The other 9 mice were used for behavioral analysis. We performed the same experiment twice for molecular and behavioral analyses and used a total of 25 mice per group (as described in Animal session). All samples were extracted the day after the last stress condition. Brain samples were immediately placed in liquid nitrogen after extraction, and kept in a −80°C freezer until later analyses.

### RNA purification

Total RNA was purified from the mouse brain using a Sepasol-RNA I Super kit (Nacalai Tesque, Kyoto, Japan) according to the manufacturer’s instructions, and treated with five units of RNase free DNase I at 37°C for 30 min to remove genomic DNA contamination. After phenol/chloroform extraction and ethanol precipitation, total RNA was dissolved in de-ionized distilled water. RNA concentrations were determined by spectrophotometry.

### Microarray analysis

We outsourced (Takara Bio Inc, Mie, Japan) the microarray analysis for the whole genome. The details were shown below.

### RNA quality check

RNA was quantified using a NanoDrop-2000 spectrophotometer and quality was monitored with the Agilent 2100 Bioanalyzer (Agilent Technologies, Santa Clara, CA).

### Label protocol (1color)

Cyanine-3 (Cy3)-labeled cRNA was prepared from 0.1 μg total RNA using the Low Input Quick Amp Labeling Kit (Agilent) according to the manufacturer’s instructions, followed by RNeasy column purification (Qiagen, Valencia, CA).

Dye incorporation and cRNA yield were checked with the NanoDrop ND-2000 Spectrophotometer.

### Hybridization protocol

A total of 0.6 μg of Cy3-labeled cRNA was fragmented at 60°C for 30 min in a reaction volume of 25 μl containing 1× Agilent fragmentation buffer and 2× Agilent blocking agent following the manufacturer's instructions.

Upon completion of the fragmentation reaction, 25 μl 2× Agilent hybridization buffer was added to the fragmentation mixture and hybridized to Agilent SurePrint G3 Mouse GE 8x60K (cat; G4858A-028005) for 17 h at 65°C in a rotating Agilent hybridization oven.

After hybridization, microarrays were washed for 1 min at room temperature with GE Wash Buffer 1 and 1 min with 37°C GE Wash buffer 2 (Agilent), then dried immediately by brief centrifugation.

### Scan protocol

Slides were scanned immediately after washing on the Agilent DNA Microarray Scanner (G2565CA) using a one color scan setting for 8×60k array slides (Scan Area 61 × 21.6 mm, Scan resolution 3 μm, the dye channel set to Green, and Green PMT set to 100%).

### Data processing

The scanned images were analyzed with Feature Extraction Software 10.10.1.1 (Agilent) using default parameters to obtain background subtracted and spatially detrended Processed Signal intensities.

### Value definition

Scaled signal intensities were adjusted to an average intensity value of 2500.

Details of the analysis method can also be found on the Gene Expression Omnibus (GEO) website. Our series entry was GSE49867 (http://www.ncbi.nlm.nih.gov/geo/query/acc.cgi?acc=GSE49867).

### Database for Annotation, Visualization, and Integrated Discovery (DAVID) web tool analysis

An approach to annotation enrichment analysis was performed using the DAVID (http://david.abcc.ncifcrf.gov/) web tools (version 6.7, 2010) [36–37]. This web-based resource provided a set of functional annotation tools for statistical enrichment of the genes categorized into Gene Ontology (GO) terms. We used the GO FAT category, which filtered out very broad GO terms to identify significantly enriched functional groups. We examined the functions of lipid metabolism, coagulation, hormonal activity, immunological function, and amine synthesis because they had a *p* value less than 0.05.

### Ingenuity Pathway Analysis (IPA)

IPA software (Ingenuity Systems, http://www.ingenuity.com) was used for microarray analyses conducted to provide functionality for the interpretation of gene expression data. The network explorer of IPA was used to detect relevant interactions among the CMS and C group genes, and identify the shortest direct paths between genes. We used this software in a similar manner to that described in previous studies [38–39].

### Quantitative real-time polymerase chain reaction (qRT-PCR)

To validate the results obtained by the microarray, DAVID, and IPA analyses, qRT-PCR was performed twice under at least 10 different experimental conditions. Total RNA (10 ng/reaction) extracted from the CMS and C groups was used in the RNA-direct SYBR Green Real-Time PCR Master Mix: One-step qPCR kit (Toyobo Co. Ltd., Tokyo, Japan). Samples were run in duplicate reactions in 96-well plates. Median threshold cycle values were used to calculate fold changes (FC) between the samples from 2 groups. FC values were normalized to glyceraldehyde-3-phosphate dehydrogenase (GAPDH) levels. The following temperature profile was used: 30 s at 90°C and 20 min at 61°C for reverse transcription according to the manufacturer’s instructions, followed by 45 cycles of 95°C for 15 s, 65°C for 15 s, and 74°C for 35 s. We used the primers of hepatocyte nuclear factor 4 alpha (Hnf4a) and GAPDH, as shown in Table 1.

**Table 1:** Structures of the primers used.

Gene	Genbank Accession		Primers (5′–3′)
GAPDH	NM_008084	sense	CCTTCCGTGTTCCTACCCCCAAT
GAPDH	NM_008084	anti-sense	TTGATGTCATCATACTTGGCAGGTTTCTC
Hnf4a	NM_008261	sense	TGATAACCACGCTACTTGCCTT
Hnf4a	NM_008261	anti-sense	AGCCTACTTCTGAATGTTTGGTGT

GAPDH, glyceraldehyde-3-phosphate dehydrogenase; Hnf4a, Hepatocyte nuclear factor 4 alpha.

### Western blotting analysis (WB)

Mouse brains were minced in Lysis buffer (80 μl) on ice and sonicated using a sonicator (Sonifier II, BRANSON). The lysate was centrifuged (12,000 rpm/13,000 G, 3 min) and the supernatant was collected. The protein concentration in the supernatant was determined with a Bio-Rad protein assay kit (Bio-Rad Laboratories). Sample buffer was added to the supernatant, and the mixture was heat-treated (95°C, 5 min). Samples were electrophoresed in a 12.5% SDS polyacrylamide gel and transferred onto polyvinylidene difluoride membranes (Hybond-P, Amersham Bioscience). Membranes were then incubated with the anti-goat Hnf4a antibody (cat no: sc-6557, Santa Cruz Biotechnology, Inc. CA, USA) overnight, washed with T-PBS, and incubated with Anti-goat IgG (cat no: sc-2020, Santa Cruz Biotechnology, Inc.). The specific binding of antibodies was captured using the LAS-4000 photo-image analyzer (Fuji Photo Film Co., Ltd.). We measured the density of stained protein bands using ImageJ (http://rsbweb.nih.gov/ij/, version 1.6) and the results obtained were normalized to GAPDH levels. We also assessed positive controls (cat no: sc-126960, Santa Cruz Biotechnology, Inc.).

### Serum data analysis

We measured triglyceride (TG), total cholesterol (T-cho), HDL cholesterol (H-cho), and cortisol levels from the sera collected. TG, T-cho, and H-cho levels were measured by enzymatic methods and cortisol by a chemiluminescent enzyme immunoassay method (CLEIA method). We outsourced (Mitsubishi Chemical Medience Co., Tokyo, Japan) the serum analysis.

### Statistical analysis

All results are expressed as the mean ± SEM. Differences between groups were analyzed by Student’s *t*-test or Mann–Whitney U-test. We used Sigmaplot (version 10.0 Systat Software, Inc., San Jose, CA, USA) for all analyses of our results.

## Results

### Behavioral tests (Reduced locomotive activity in CMS mice)

**Fig 1:**
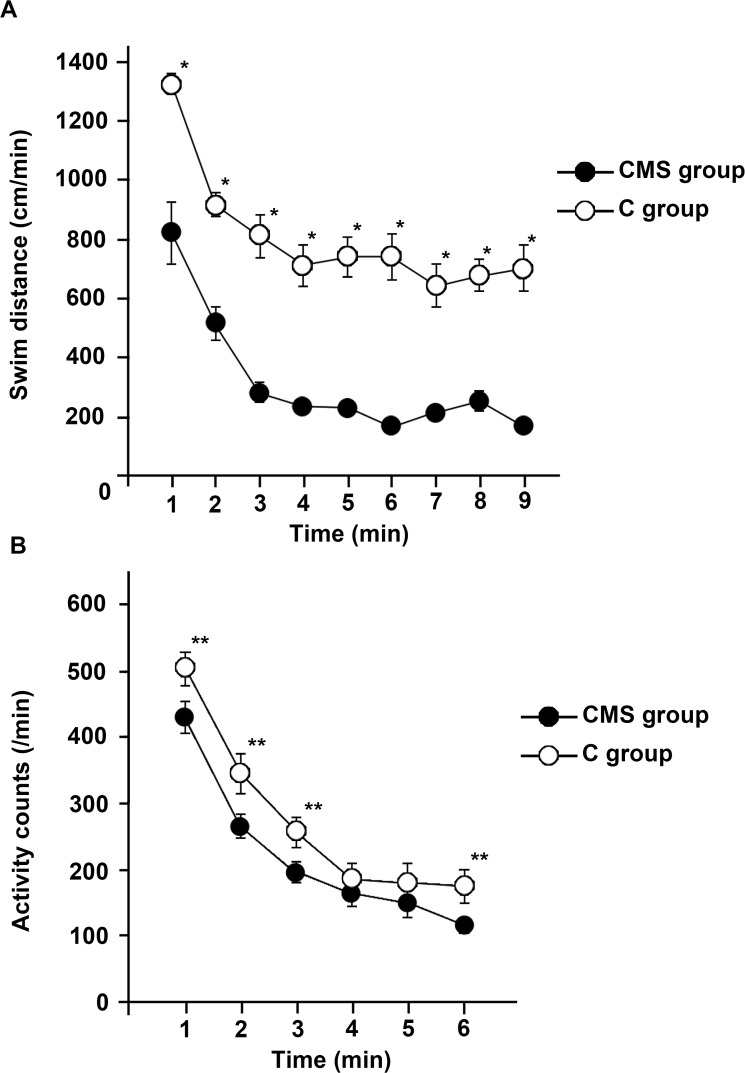
CMS group displayed depressive symptom patterns in the behavioral tests. (A) Open space swimming test (OST). Mice performed the OST following exposure to CMS. The swimming distance was measured by recording movement with a digital video camera and analyzed using a computerized video-based tracking system. The results are expressed as the average values obtained from the CMS and C groups, and were analyzed by Student’s *t*-test (*n* = 9 for each group; *: *p*<0.001). (B) Tail suspension test (TST). The activity counts of the CMS group were measured using the Be-sensor system, which analyzed radiated heat energy. The mean values in CMS and C groups are presented. The activity counts of the CMS group were significantly lower than those of the controls in the first 3 minutes and the last minute of the test. The data were analyzed by Student’s *t*-test and a *p* value less than 0.05 was considered significant (*n* = 9 per each group; **: *p*<0.05).

The results of the OST and TST are shown in Fig. 1. A marked reduction in the swimming speed was observed until 2 to 3 min after the start of swimming and was maintained thereafter in both the CMS and C groups (Fig. 1A). The mean swimming distance was significantly shorter in the CMS group than in the C group at each time point. Physical activity, including heat energy radiated in the TST, was significantly lower in the CMS group (Fig. 1B). Therefore, locomotive activity was reduced in CMS mice. No significant differences were observed in weight changes between the two groups (data not shown).

### Microarray analysis of the PFC

Because mice exposed to CMS exhibited behavioral patterns consistent with those observed in MDD patients, we subsequently performed microarray analysis on the PFC to compare gene expression profiles of the CMS and C group. We isolated a total of 494 genes in CMS group whose expression was more than 2-fold higher than or less than half that of the C group. We searched for the functions, categories, and interactions of these genes by DAVID and IPA.

### DAVID analysis (Categorization of CMS-related genes)

We categorized the 494 genes by DAVID according to their biological functions. We selected genes whose functions were related to lipid metabolism, hormonal activity, coagulation, immunological function, and amine synthesis. As shown as Table 2 and S1 Table, 41 genes were related to lipid metabolism, 22 to hormonal activity, 17 to coagulation, 77 to immunological function and 13 to amine synthesis, respectively. Some genes overlapped the different categories. We analyzed the relationships between these genes by IPA.

**Table 2:** Classification and enrichment of CMS genes extracted from DAVID results.

Group	GO category	Count	*p* value
Lipid metabolism	GO:0006869 lipid transport	19	< 0.001
	GO:0010876 lipid localization	19	< 0.001
	GO:0055088 lipid homeostasis	7	0.002
	GO:0006638 neutral lipid metabolic process	8	0.002
	GO:0006641 triglyceride metabolic process	7	0.002
	GO:0008289 lipid binding	23	0.025
Hormonal activity	GO:0008202 steroid metabolic process	23	< 0.001
	GO:0009725 response to the hormone stimulus	19	< 0.001
	GO:0003707 steroid hormone receptor activity	6	0.048
Coagulation	GO:0050817 coagulation	20	< 0.001
	GO:0007596 blood coagulation	20	< 0.001
Immunological function	GO:0006954 inflammatory response	33	< 0.001
	GO:0006952 defense response	48	< 0.001
	GO:0050778 positive regulation of immune response	24	< 0.001
	GO:0002253 activation of immune response	19	< 0.001
	GO:0019724 B cell mediated immunity	14	< 0.001
	GO:0002449 lymphocyte mediated immunity	15	< 0.001
	GO:0002252 immune effector process	19	< 0.001
	GO:0006955 immune response	40	< 0.001
	GO:0034097 response to cytokine stimulus	9	< 0.001
	GO:0002697 regulation of immune effector process	10	0.007
Amine synthesis	GO:0009310 amine catabolic process	9	0.003
	GO:0009309 amine biosynthetic process	9	0.007

CMS, chronic mild stress; GO, Gene Ontology; DAVID, the Database for Annotation, Visualization, and Integrated Discovery.

### IPA analysis

**Fig 2:**
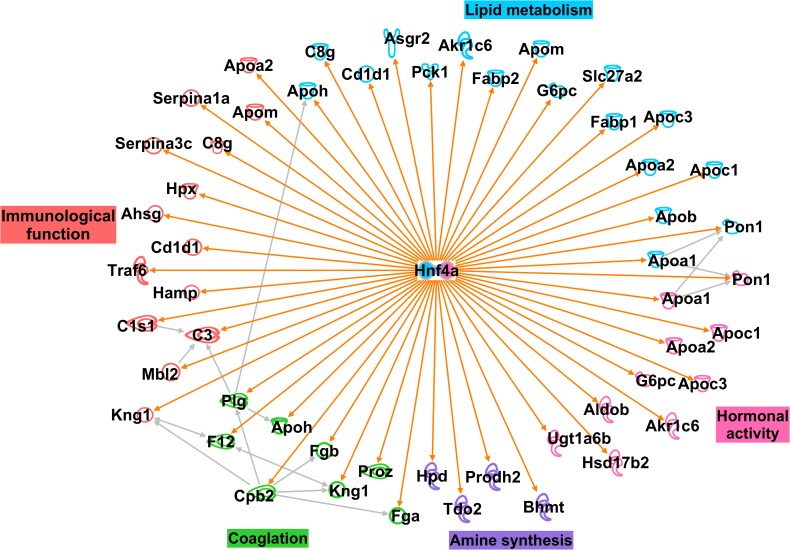
IPA analysis; direct networks among Hnf4a and other genes related to homeostasis and amine synthesis. Total mRNA was extracted from the PFC of the CMS and C groups and analyzed by microarray. A number of genes were selected from the genes differentially expressed in the PFC of the CMS groups, and transferred to DAVID web tool analysis. Hnf4a had an impact on these genes. They were categorized into genes related to lipid metabolism (blue), hormonal activity (green), coagulation (pink), immunological function (red), and amine synthesis (purple). The details of these genes including their log_2_ ratios are shown in Table 3.

We previously categorized all of the isolated genes. We hypothesized that some candidate genes were augmented in the PFC and directly responsible for the disturbances reported in lipid metabolism, hormonal activity, coagulation and immunological function, under a depressive state. We searched for only direct interactions among extracted genes from DAVID analysis using IPA. As shown in Fig. 2, we identified one gene, ‘Hnf4a’ that had direct interactions between the extracted genes and was located in the center of these interactions. Hnf4a was categorized into ‘steroid hormone receptor activity’ and ‘lipid binding’ by DAVID analysis. Furthermore, Hnf4a had a strong influence on genes that were related to lipid metabolism, hormonal activity, coagulation, immunological function, and amine synthesis. The gene details and log_2_ ratio of IPA are shown in Table 3. The results of the core analyses for Hnf4a are also in S2 Table.

**Table 3:** Gene details and differences observed between the CMS and control groups with the IPA analysis.

Group	Genes	Entrez Gene Name	Log_2_ Ratio
Lipid metabolism	Akr1c6	aldo-keto reductase family 1, member C4	3.08
	Apoa1	apolipoprotein A-I	3.98
	Apoa2	apolipoprotein A-II	3.12
	Apob	apolipoprotein B (including Ag(x) antigen)	3.79
	Apoc1	apolipoprotein C-I	3.18
	Apoc3	apolipoprotein C-III	2.85
	Apoh	apolipoprotein H (beta-2-glycoprotein I)	2.89
	Apom	apolipoprotein M	2.31
	Asgr2	asialoglycoprotein receptor 2	1.31
	C8g	complement component 8, gamma polypeptide	1.44
	Cd1d	CD1d molecule	1.12
	Fabp1	fatty acid binding protein 1, liver	3.35
	Fabp2	fatty acid binding protein 2, intestinal	1.53
	Hnf4a	hepatocyte nuclear factor 4, alpha	3.56
	Pck1	phosphoenolpyruvate carboxykinase 1 (soluble)	3.58
	Slc27a2	solute carrier family 27 (fatty acid transporter), member 2	1.08
Hormonal activity	Aldob	aldolase B, fructose-bisphosphate	2.54
	G6pc	glucose-6-phosphatase, catalytic subunit	2.68
	Hsd17b2	hydroxysteroid (17-beta) dehydrogenase 2	1.82
	Pon1	paraoxonase 1	3.36
	Ugt1a6b	UDP glucuronosyltransferase 1 family, polypeptide A6	1.48
Coagulation	Cpb2	carboxypeptidase B2 (plasma)	2.36
	F12	coagulation factor XII (Hageman factor)	1.62
	Fga	fibrinogen alpha chain	1.13
	Fgb	fibrinogen beta chain	3.25
	Kng1	kininogen 1	3.00
	Plg	plasminogen	3.08
	Proz	protein Z, vitamin K-dependent plasma glycoprotein	2.34
Immunological function	Ahsg	alpha-2-HS-glycoprotein	3.39
	C1s1	complement component 1, s subcomponent	1.73
	C3	complement component 3	2.90
	Hamp	hepcidin antimicrobial peptide	2.81
	Hpx	hemopexin	2.97
	Mbl2	mannose-binding lectin (protein C) 2	3.06
	Serpina1a	serine peptidase inhibitor, clade A, member 1A	3.07
	Serpina3c	serine peptidase inhibitor, clade A, member 3c	3.16
	Traf6	TNF receptor-associated factor 6	-1.46
Amine synthesis	Bhmt	betaine—homocysteine S-methyltransferase	2.21
	Hpd	4-hydroxyphenylpyruvate dioxygenase	3.57
	Prodh2	proline dehydrogenase (oxidase) 2	1.66
	Tdo2	tryptophan 2,3-dioxygenase	2.60

CMS, chronic mild stress; IPA, Ingenuity Pathway Analysis.

### qRT-PCR

At first, to verify these results of DAVID and IPA, we selected 10 genes including selected by these analyses, performed 10 qRT-PCR experiments (S3 Table), and compared the results with those obtained from microarray analyses using Spearman's rank correlation tests. The results supported the significant correlation between qRT-PCR and microarray analyses, showing an rs value of 0.903 with a two-tailed *p* value of less than 0.001 (S4 Table).

**Fig 3:**
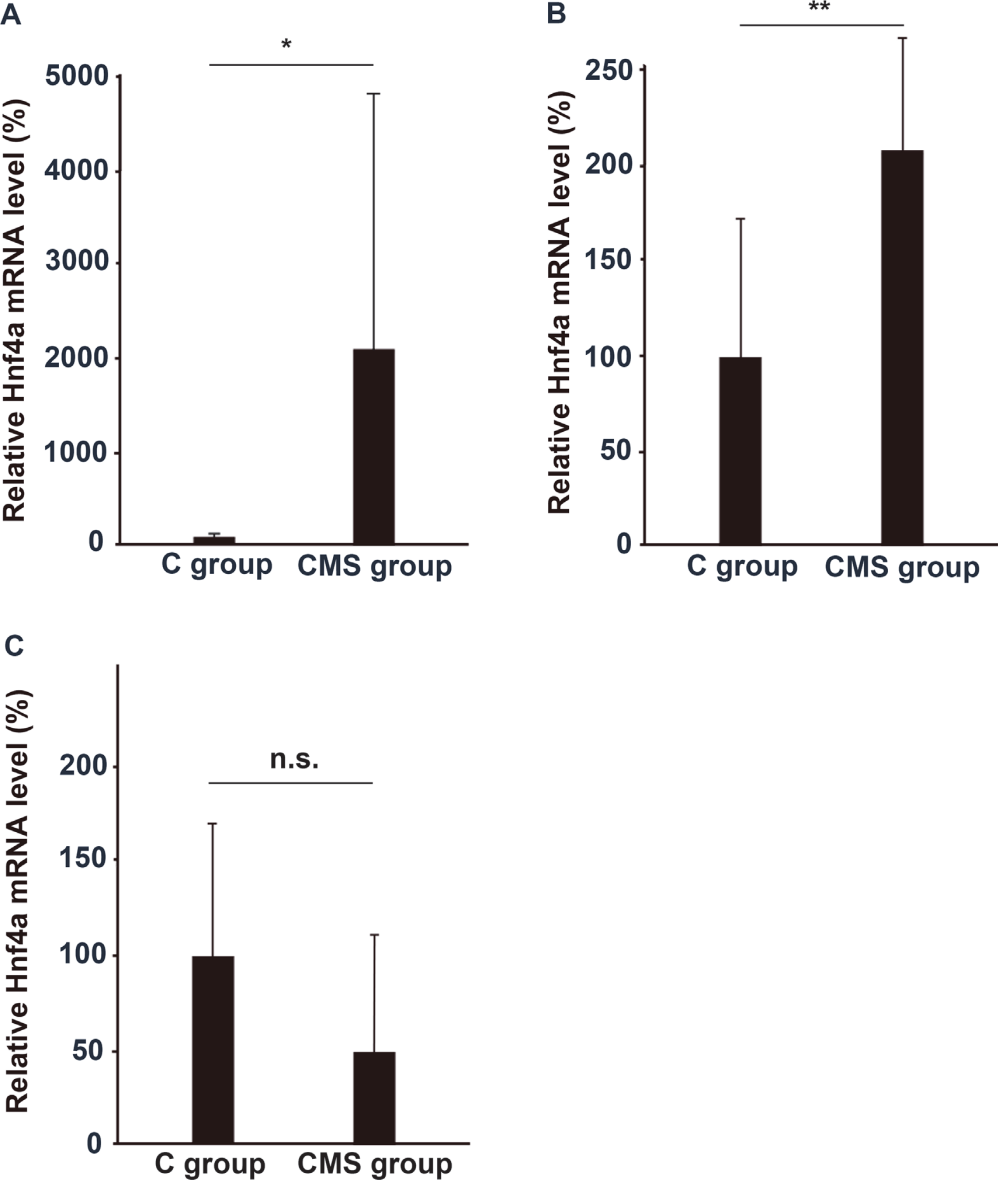
Enhanced expression of Hnf4a mRNA in the PFC, thalamus, and hippocampus of the CMS group. We performed qRT-PCR analysis on Hnf4a mRNA expressed in the (A) PFC (*n* = 10), (B) thalamus (*n* = 7), and (C) hippocampus (*n* = 7) of the CMS group. The mean concentration of mRNA from the brains of the C group was set at 100%, and relative mRNA levels in each part of the brain were expressed by the mean multiplicity of the CMS group. The results of the PFC were analyzed with the Mann–Whitney U test, and those of the thalamus and hippocampus were analyzed with Student’s *t*-test (*: *p*<0.005, **: *p*<0.05, n.s.: not significant).

Subsequently, we confirmed the expression of Hnf4a in the PFC (Fig. 3A). Hnf4a expression in the PFC was significantly higher in the CMS group than in the C group, which confirmed the results of microarray analysis. We also examined the expression of Hnf4a in the thalamus, and hippocampus. Hnf4a mRNA expression was significantly increased in the thalamus, but was not in the hippocampus (Fig. 3B and C). Thus, Hnf4a expression was significantly increased in the PFC and thalamus under a depressive state.

### WB

**Fig 4:**
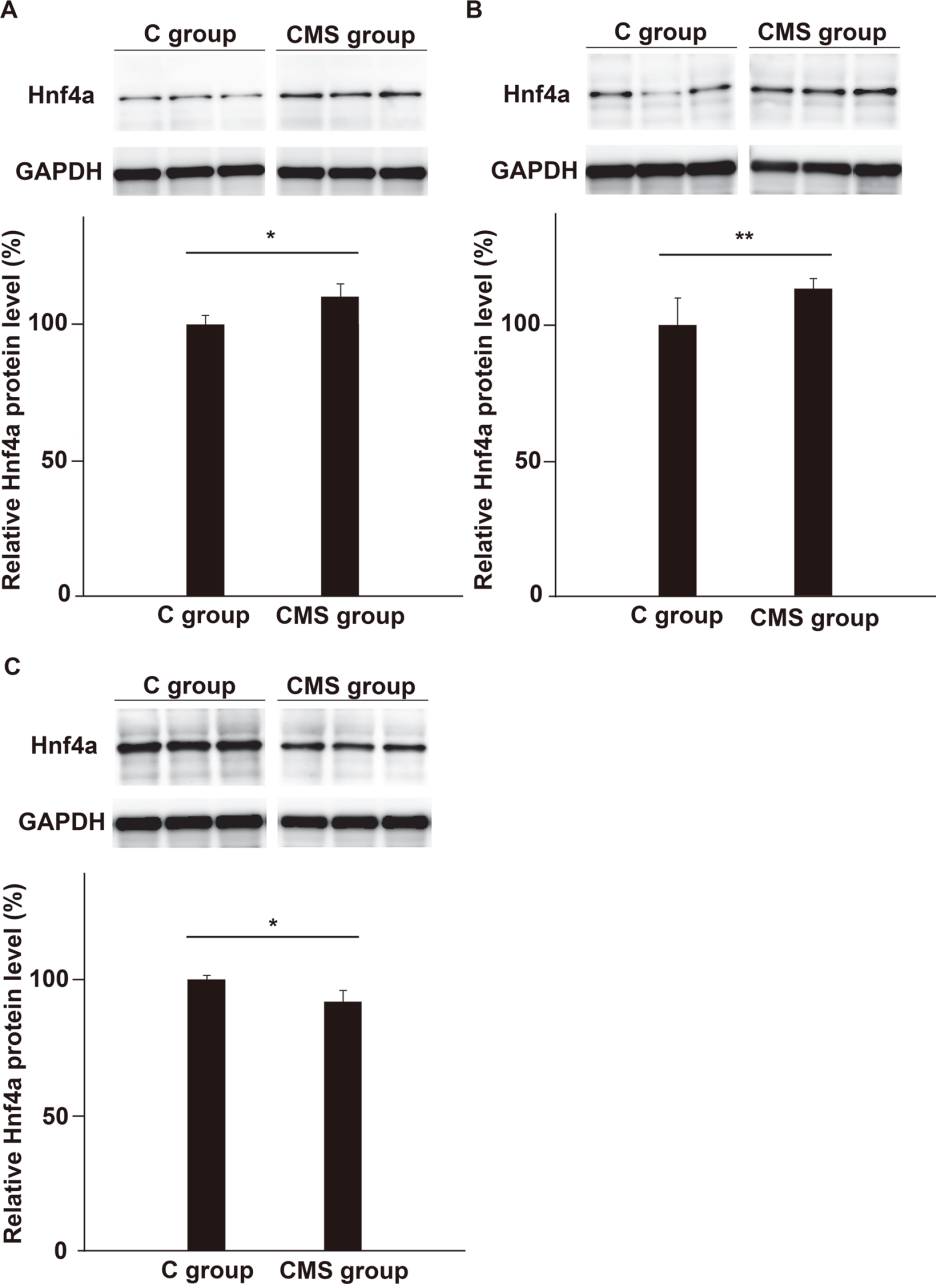
Expression of the Hnf4a protein in the brains of CMS and C groups. Protein extracts were prepared from the PFC (A), thalamus (B), and hippocampus (C) from the CMS and C groups. The density of the stained protein dots of Hnf4a were normalized to that of GAPDH. We clearly demonstrated that the Hnf4a protein was expressed in the brain (S1 Fig.). Expression of the Hnf4a protein in the PFC and thalamus was significantly higher in the CMS group than in the control group. On the other hand, its expression was reduced in the hippocampus. (A, B) We examined the expression of the Hnf4a protein in the PFC and thalamus of the CMS group by WB, and significant differences were observed between the CMS and C groups. WB results of the PFC and thalamus were analyzed using Student’s *t*-test (A: *n* = 5 for each group, B: *n* = 6 for each group). (C) The same analysis was performed on the hippocampus by WB. Significantly lower Hnf4a expression was found in the hippocampus of the CMS group. The data were analyzed by Student’s *t*-test and the *p* value obtained was 0.007 (*n* = 6 for each group; *: *p*<0.01, **: *p*<0.05).

We further examined augmented expression of Hnf4a in the PFC of CMS mice by WB analysis of the protein levels produced (Fig. 4A). In accordance with the results of qRT-PCR analysis, quantitative analysis of the representative blots indicated the enhanced synthesis of the Hnf4a protein in the PFC of CMS mice. Hnf4a protein expression in the PFC was higher in the CMS group than in the C group (Fig. 4A). Similar to the results for mRNA levels in the thalamus and hippocampus, synthesis of the Hnf4a protein was higher in the thalamus and lower in the hippocampus in the CMS group than in the C group (Fig. 4B, 4C). We checked that there were no non-specific bands and also confirmed the bands observed using a positive control, as shown in S1 Fig.

### Serum data

Metabolic parameters as well as circulating hormones were measured because they were previously shown to be altered in association with a depressive state. The results obtained are shown in Table 4. Although no significant differences were observed in T-cho or H-cho levels between the groups, TG levels were significantly higher and cortisol levels were significantly lower in the CMS group than in the C group.

**Table 4:** TG, T-cho, H-cho, and cortisol levels in the serum.

	CMS group (*n* = 15)		C group (*n* = 15)			
	Mean	SD	Mean	SD	t	*p* value
TG (mg/dl)	52.4	13.8	43.0	8.8	2.23	0.034
T-cho (mg/dl)	88.2	9.9	87.3	13.6	0.21	0.838
H-cho (mg/dl)	67.0	10.5	64.3	14.4	-0.038	0.72
Cortisol (μg/dl)	0.90	0.09	1.39	0.41	-3.58	0.001

CMS group, chronic mild stress group; C group, control group; TG, triglyceride; T-cho, total cholesterol; H-cho, HDL-cholesterol; SD, standard deviation.

Serum cytokines levels were also measured to examine group differences in inflammatory responses. The inflammatory cytokines IL-5, IL-12 beta, IL-17 alpha, and Tnf-alpha were significantly higher in the CMS group than the C group (S5 Table).

## Discussion

The results of the present study that have crucial implications regarding the pathophysiology of MDD were: 1) the CMS model clearly engendered a set of behaviors indicative of depressive symptoms observed in humans, 2) the CMS model animals were significantly more likely than the controls to show lipid metabolic dysfunction, especially hypertriglycemia, 3) the presence of the Hnf4a protein was confirmed in 3 parts of the brain: the PFC, thalamus, and hippocampus, and 4) Hnf4a may have affected molecules related to lipid metabolism, hormonal activity, coagulation, immunological function, or amine synthesis under a depressive state, indicating that Hnf4a may play a role in the interactions between MDD and dysfunctions of physiological homeostasis.

The serum level of inflammatory cytokines such as Tnf-alpha may increase under depressive conditions, and their elevated levels may trigger MDD [12, 18]. Metabolic syndrome, characterized by increased TG levels and non-treated Diabetes Mellitus (DM), may also increase the risk of MDD [28, 40]. Conversely, MDD has been regarded as a risk factor for the development of hyperlipidemia [27]. Significantly greater elevations in inflammatory cytokine and TG levels were observed in the CMS group, which suggests the possibility that unpredictable and chronic stress may lead to inflammation and dyslipidemia in our model.

Hnf4a is a transcription factor that is involved in gluconeogenesis and lipid homeostasis [41], and may be closely related to the development of DM in the young [42]. In the present study, we confirmed that Hnf4a was clearly up-regulated in the PFC and thalamus and down-regulated in the hippocampus in CMS mice.

A number of isolated genes were detected in the present study using microarray analysis. DAVID categorized these genes into those controlling lipid metabolism, coagulation, hormonal activity, immunological function, and amine synthesis, with significant interactions among each categorized gene. The PFC is a projection area for dopaminergic and serotonergic neurons and modulates these systems [43–45]. For instance, Tryptophan 2,3-dioxygenase (Tdo2) was categorized as an amine synthesis molecule by DAVID in this study, and it is a rate-limiting enzyme expressed in the pathway from tryptophan to kynurenine, which is an alternative pathway to the tryptophan-serotonin pathway [15]. When stimulated by stress, Tdo2 reduces serotonin levels because of a shift in tryptophan metabolism from serotonin to kynurenine [15]. We found higher expression of Tdo2 in the CMS group than the C group, which may have led to higher depression-like behaviors in the CMS group because of deficient serotonin levels through regulation by Tdo2. Furthermore, Hnf4a increased the expression of Tdo2, and the augmentation of Tdo2 expression may be related to both stress and elevated Hnf4a levels [15, 46]. Hnf4a itself was identified to have a regulatory role in lipid metabolism by our core analysis. Indeed, seventeen genes associated with lipid metabolism were regulated by Hnf4a in our analysis. Hnf4a also increased insulin resistance, DM, glucose tolerance, and inflammatory responses. It also appears to affect coagulation, hormonal activity and immunological functions, as 8, 10 and 14 molecules related to these processes were affected by Hnf4a, respectively.

Recently, there has been a growing interest in the role of Hnf4a in humans. For instance, the Tnf antagonist infliximab was found to be effective in ameliorating depressive symptoms, and when patients experiencing high inflammation were administered infliximab, they showed greater reductions in depressive symptoms than patients treated with placebo [41, 47]. Additionally, infliximab also affects gluconeogenesis and lipid homeostasis through Hnf4a [41]. These results seem to support our speculation that Hnf4a might exert direct effects on the levels of inflammatory cytokines including Tnf-alpha, along with its effects on lipid metabolism.

Thus, Hnf4a may have critical effects in monoamine deficiency and impaired lipid metabolism, coagulation, hormonal activity, and immunological functions in depression.

The thalamus is generally considered to regulate body homeostasis, hormone levels, and food intake [7, 48]. In this study, similar results were observed in the PFC and thalamus, which suggests that Hnf4a may also exert some effects on homeostasis, especially in the regulation of cortisol levels. As shown in Fig. 2, Hnf4a regulated hormonal activity genes. Our results suggest the possibility that the up-regulation of Hnf4a expression in stressful situations may stimulate the activity of steroid hormone receptors.

However, the results of the present study demonstrate that Hnf4a may be down-regulated in the hippocampus. Neurogenesis in the hippocampus of mammals may be significantly less in depressive status than in non-depressive healthy controls, which is consistent with the findings obtained from adult humans [49–50]. Neurogenesis may also be decreased in the hippocampus and apoptosis may be enhanced in cerebral cells of CMS-treated rats [30]. Our results show that expression of the Hnf4a protein in the hippocampus was lower in the CMS group than in the C group. Liver cells have also been shown to be more susceptible to lipopolysaccharide-mediated apoptosis in Hnf4a knock-out mice [51]. In addition, Hnf4a knock-out mice developed severe hepatomegaly and steatosis, resulting in premature death [52]. Based on these findings, Hnf4a can be considered to be an essential transcription factor that protects against cell apoptosis, and the down-regulation of Hnf4a expression may lead to apoptosis and the inhibition of neurogenesis in the hippocampus.

In the present study, we only measured Hnf4a in 3 brain regions. To more thoroughly examine our hypotheses, especially those related to the central regulation of physiological functions such as lipid metabolism, coagulation, hormonal activity, immunological function, and amine synthesis, future studies should analyze metabolism in peripheral organs to clarify the interactions and global mechanisms of Hnf4a function. Moreover, the effects of antidepressant drugs on the expression of Hnf4a and other molecules should be examined in our model. Further studies to measure the role and dynamics of Hnf4a under a depressive state, including hormonal metabolism and its distribution in depressed humans, are warranted. Finally, our suggestions regarding the crucial role of Hnf4a on physiological homeostasis under depressive status are based on web-based informatics using DAVID. We used DAVID as it is the most suitable and reliable method at present for elucidating the molecular interactions between MDD and the dysregulation of physiological homeostasis. Thus its reliability should be further examined once more advanced methods become available.

In conclusion, in the present study, we demonstrate the possibility that Hnf4a may be a central regulator of genes involved in lipid metabolism, hormonal activity, coagulation, immunological function, and amine synthesis. The Hnf4a protein was clearly expressed in the brain, and this gene may be both essential for life and have a critical impact on physiological homeostasis under depressive status. Regardless of the limitations of this study, our finding should provide valuable insights into the mechanisms responsible for the interactions between MDD and physiological homeostasis in laboratory animals and human patients.

## Supporting Information

S1 Fig Western blotting using 2 different antibodies clearly detected the Hnf4a protein in the brain. We used 2 different antibodies (A): a goat polyclonal anti-mouse Hnf4a antibody (cat no: sc-6557, Santa Cruz Biotechnology, Inc.) and (B): a mouse monoclonal Hnf4a antibody (cat no: PP-K9218-00, Perseus Proteomics, Inc.) for western blotting analysis to detect Hnf4a in the brain. We used the thalamus lysate and 293T lysate (cat no: sc-126960, Santa Cruz Biotechnology, Inc.) as positive controls (PC), to confirm that we successfully stained the Hnf4a protein in the brain. We also examined these same thalamus samples with only secondary antibodies; (C) anti-goat IgG, and (D) anti-mouse IgG. No bands were observed.
(TIF)

S1 Table Functional annotation clustering of gene differences between the CMS and C groups based on DAVID analysis. (XLSX)

S2 Table Core analyses of Hnf4a using IPA. We performed the core analyses using IPA. We extracted the functions and the diseases related to Hnf4a from all results of the core analyses.
(XLSX)

S3 Table The primers for the validation of microarray data of the PFC with qRT-PCR one. We confirmed the expression of 10 genes from IPA results, including the genes shown in Fig. 2, by qRT-PCR. We showed the primers used for qRT-PCR experiments. PFC, prefrontal cortex; qRT-PCR, quantitative real-time polymerase chain reaction;
(DOCX)

S4 Table Validation of microarray data of the PFC with qRT-PCR one. We compared the results of microarray from qRT-PCR to determine significant correlations by Spearman’s rank collection test, which revealed a significant correlation (rs = 0.903, p<0.001). PFC, prefrontal cortex; qRT-PCR, quantitative real-time polymerase chain reaction; FC(qRT-PCR), fold change based on the results obtained with qRT-PCR; FC(Microarray), fold change based on the results obtained by microarray.
(DOCX)

S5 Table Levels of IL-5, IL-12b, IL-17A, and TNF-a in the serum. To investigate the influence of immune factors to MDD, we measured the serum cytokines by Bio-Plex Pro Mouse Cytokine 23-Plex Panel (cat; M60-009RDPD, Bio-Rad Laboratories, Inc.) and Bio-Plex Pro Mouse Cytokines GII 9-Plex Panel (cat; MD0-00000EL). The cytokine in the CMS group that exhibited significantly higher levels than in the C group were IL-5, IL-12b, IL-17A, and TNF-a. Conversely, the levels of IL-1b, IL-2, IL-6, IL-9, IL-10, and IL-18 were not significantly different between the groups (data not shown). CMS group, chronic mild stress group; C group, control group; IL-5, interleukin 5; IL-12b, interleukin 12 beta; IL-17A, interleukin 17 alpha; TNF-a, tumor necrosis factor alpha; SD, standard deviation.
(DOCX)
