# Supplementary figures and images for: Hepatocyte Nuclear Factor 4 Alpha Is a Key Factor Related to Depression and Physiological Homeostasis in the Mouse Brain
Source: PLoS One. 2015 Mar 16;10(3):e0119021. doi: 10.1371/journal.pone.0119021 (PMC4361552; doi:10.1371/journal.pone.0119021)

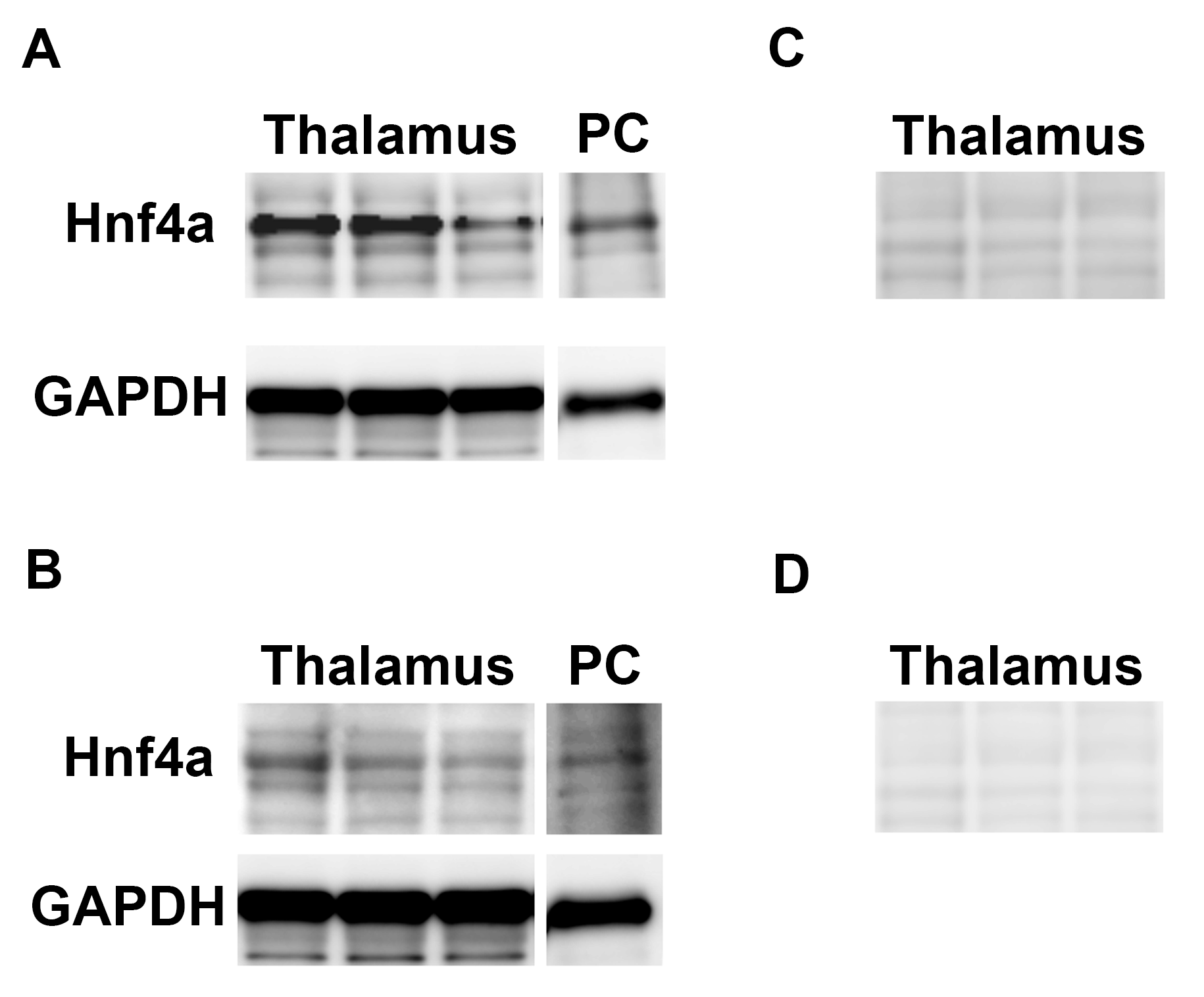

Supplement: S1 Fig — We used 2 different antibodies (A): a goat polyclonal anti-mouse Hnf4a antibody (cat no: sc-6557, Santa Cruz Biotechnology, Inc.) and (B): a mouse monoclonal Hnf4a antibody (cat no: PP-K9218-00, Perseus Proteomics, Inc.) for western blotting analysis to detect Hnf4a in the brain. We used the thalamus lysate and 293T lysate (cat no: sc-126960, Santa Cruz Biotechnology, Inc.) as positive controls (PC), to confirm that we successfully stained the Hnf4a protein in the brain. We also examined these same thalamus samples with only secondary antibodies; (C) anti-goat IgG, and (D) anti-mouse IgG. No bands were observed. (TIF) [file pone.0119021.s001.tif]
