# Supplementary material for: Hepatocyte Nuclear Factor 4 Alpha Is a Key Factor Related to Depression and Physiological Homeostasis in the Mouse Brain
Source: PLoS One. 2015 Mar 16;10(3):e0119021. doi: 10.1371/journal.pone.0119021 (PMC4361552; doi:10.1371/journal.pone.0119021)
Supplement: S3 Table — We confirmed the expression of 10 genes from IPA results, including the genes shown in Fig. 2, by qRT-PCR. We showed the primers used for qRT-PCR experiments. PFC, prefrontal cortex; qRT-PCR, quantitative real-time polymerase chain reaction; (DOCX) [file pone.0119021.s004.docx]

| Primers used for qRT-PCR experiments | | |
| --- | --- | --- |
| Gene symbol | Forward primer (5'-3') | Reverse primer (5'-3') |
| Aldob | ATCCTTTTCCACGAGACCCT | TGATGCCCACCACAATTCCC |
| Arc | AGGAGACCATCGCCAACCTG | GCCCACCTCTCCAGACGGTA |
| Slc27a2 | TGAACTGCTTCGGTACCTGT | ACACATCTCCTCGTAAGCCAT |
| Asgr2 | AGCCAGTTTCATATTTGGATAGGTCT | GGCCCAATTCCTGTAGTTGCTT |
| Btg2 | TAGATAGGAGCCACCCGACCC | AAAATACAGTTCCCCAGGTTGAGG |
| S100a9 | GCAGCATAACCACCATCATCGAC | CTGTGCTTCCACCATTTGTCTGA |
| Kng1 | AGCACCTTGGACAAAGTCTCG | TCTTGCCATTTCAGTCATATCTAATGCTT |
| Ahsg | CATAAAGCCAGCAGCAACACT | AGAGCACCTTTCAGAGTCGT |
| Cpb2 | TGACATGCTCCAGAAAATCTACATCGG | ACAGTCGATCCAGATGGCATT |
| Proz | CTCACCGCAGTCCCGAGA | TGTGACCGGATTCGACCCTC |
