# Supplementary material for: Hepatocyte Nuclear Factor 4 Alpha Is a Key Factor Related to Depression and Physiological Homeostasis in the Mouse Brain
Source: PLoS One. 2015 Mar 16;10(3):e0119021. doi: 10.1371/journal.pone.0119021 (PMC4361552; doi:10.1371/journal.pone.0119021)
Supplement: S4 Table — We compared the results of microarray from qRT-PCR to determine significant correlations by Spearman’s rank collection test, which revealed a significant correlation (rs = 0.903, p<0.001). PFC, prefrontal cortex; qRT-PCR, quantitative real-time polymerase chain reaction; FC(qRT-PCR), fold change based on the results obtained with qRT-PCR; FC(Microarray), fold change based on the results obtained by microarray. (DOCX) [file pone.0119021.s005.docx]

| DATA used for Spearman's correlation test | | | |
| --- | --- | --- | --- |
| GenBank accession | Gene symbol | FC （qRT-PCR） | FC （Microarray） |
| NM_144903 | Aldob | 6.666 | 5.816 |
| NM_018790 | Arc | 0.294 | 0.308 |
| NM_011978 | Slc27a2 | 2.509 | 2.114 |
| NM_007493 | Asgr2 | 7.543 | 2.479 |
| NM_007570 | Btg2 | 0.425 | 0.441 |
| NM_009114 | S100a9 | 1.605 | 2.297 |
| NM_023125 | Kng1 | 12.367 | 7.974 |
| NM_013465.2 | Ahsg | 11.738 | 10.483 |
| NM_019775.3 | Cpb2 | 5.588 | 5.134 |
| AK005011 | Proz | 4.930 | 5.063 |
