# Supplementary material for: Hepatocyte Nuclear Factor 4 Alpha Is a Key Factor Related to Depression and Physiological Homeostasis in the Mouse Brain
Source: PLoS One. 2015 Mar 16;10(3):e0119021. doi: 10.1371/journal.pone.0119021 (PMC4361552; doi:10.1371/journal.pone.0119021)
Supplement: S5 Table — To investigate the influence of immune factors to MDD, we measured the serum cytokines by Bio-Plex Pro Mouse Cytokine 23-Plex Panel (cat; M60-009RDPD, Bio-Rad Laboratories, Inc.) and Bio-Plex Pro Mouse Cytokines GII 9-Plex Panel (cat; MD0-00000EL). The cytokine in the CMS group that exhibited significantly higher levels than in the C group were IL-5, IL-12b, IL-17A, and TNF-a. Conversely, the levels of IL-1b, IL-2, IL-6, IL-9, IL-10, and IL-18 were not significantly different between the groups (data not shown). CMS group, chronic mild stress group; C group, control group; IL-5, interleukin 5; IL-12b, interleukin 12 beta; IL-17A, interleukin 17 alpha; TNF-a, tumor necrosis factor alpha; SD, standard deviation. (DOCX) [file pone.0119021.s006.docx]

|  | CMS group (n = 9) | | C group (n = 9) | |  |  |
| --- | --- | --- | --- | --- | --- | --- |
|  | Mean | SD | Mean | SD | t | *p* value |
| IL-5 (pg/ml) | 27.3 | 4.53 | 22.6 | 2.5 | 2.32 | 0.039 |
| IL-12b (pg/ml) | 647.8 | 69.9 | 837.6 | 116.5 | -3.42 | 0.007 |
| IL-17A (pg/ml) | 65.3 | 9.1 | 46.4 | 10 | 3.55 | 0.005 |
| TNF-a (pg/ml) | 190.0 | 6.8 | 161.0 | 16.9 | 2.82 | 0.023 |
